# Temporal Dynamics of Biological Invasions: Perception of Host Quality Differs Between Native and Alien Host Species

**DOI:** 10.1002/ece3.72270

**Published:** 2025-10-06

**Authors:** Dariusz Halabowski, Abhishek Nair Anil, Grzegorz Zięba, Kacper Pyrzanowski, Joanna Grabowska, Carl Smith, Martin Reichard

**Affiliations:** ^1^ Faculty of Biology and Environmental Protection, Department of Ecology and Vertebrate Zoology University of Lodz Lodz Poland; ^2^ Institute of Vertebrate Biology of the Czech Academy of Sciences Brno Czech Republic; ^3^ Faculty of Science, Department of Botany and Zoology Masaryk University Brno Czech Republic

**Keywords:** affiliate species, behavioural decision, coevolutionary dynamics, freshwater mussels, parasitism, reproductive behaviour, species interactions

## Abstract

The spread of non‐native species into new regions is a dynamic process driven by behavioural adaptations to local environments and species interactions. Interactions between coexisting populations can lead to localised coevolutionary patterns, shaped by the duration of their co‐occurrence. We investigated the relationship between the European bitterling fish ( *Rhodeus amarus* ), a parasitic fish that lays eggs in the gills of unionid mussels, and an invasive mussel host, *Sinanodonta woodiana* , which has spread across Europe over the past 50 years. The bitterling, a host generalist, can parasitise any European unionid mussel species, but its reproductive success with *S. woodiana* is limited due to the mussel's ability to reject bitterling eggs. We tested three hypotheses on the role of rapid local coevolution in host choice using *S. woodiana* populations with recent (5 years), intermediate (17 years), and long‐term (40+ years) associations with European bitterling. We experimentally evaluated the spawning preferences of four bitterling populations: three coexisting with their respective *S. woodiana* populations and one naïve to *S. woodiana* . All bitterling populations avoided *S. woodiana* from the most recently established population. Neither local coexistence nor individual mussel quality influenced bitterling responses to *S. woodiana* . In contrast, bitterling selected native *Anodonta anatina* mussels based on individual quality rather than population identity. These findings suggest that native species can recognise and avoid an invasive host at the invasion front, where co‐occurrence is recent.

## Graphical abstract

The paper uses a set of parasite and host populations to test how time since invasion affects the behavioural interactions between the parasite—the European bitterling ( *Rhodeus amarus* ) and their hosts—freshwater mussels. It compares how bitterling make decisions regarding oviposition into the invasive species, Chinese pond mussel, *Sinanodonta woodiana* , and contrasts it with their decision towards a native host mussel, *Anodonta anatina* . In short, our results suggest that a host at an invasion front (with recent co‐occurrence with local species) can be recognized by native species and negatively discriminated.

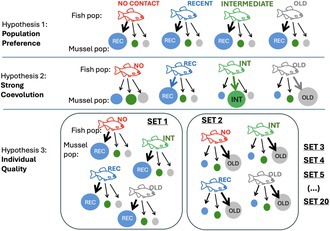

## 1 Introduction

Human‐mediated introductions of alien (non‐native) species far beyond their native distributions have led to the successful establishment and dispersal of thousands of species across new ranges (Vilà et al. 2009). Biological invasions result in the local extinction or significant decline of rare and endemic species, alterations in community structure, homogenisation of flora and fauna in previously isolated areas, loss of genetic integrity through hybridisation, and changes in habitat (Hulme 2007; Simberloff et al. 2013). These consequences make biological invasions one of the primary drivers of global biodiversity change (Hobbs et al. 2006; Sage 2020). However, only a fraction of alien species have become truly invasive, while the impacts of others remain more subtle.

The expansion of alien species is a dynamic process. It typically begins with an initial lag phase during which the abundance of an alien species remains low, and its population growth rate and impacts are minor and relatively unnoticed (Crooks 2005). The success or failure of establishment depends on propagule pressure (Lockwood et al. 2005) and invader traits, but also on the properties of the recipient community, such as complexity and resistance (Levine and D'Antonio 1999; Kolar and Lodge 2001; Whitney and Gabler 2008). The newcomer species must integrate into existing interactions to find a suitable niche space or risk elimination by local predators, parasites, or competitors. This biological filter determines whether the alien species establishes self‐sustaining populations, increases growth rates, and expands its invasive range (Pearson et al. 2018). The invader initially exploits naïve prey and avoids recognition by local enemies (David et al. 2017), but reciprocal recognition develops over time. This process is facilitated if the invader has close relatives among native species (Gallien and Carboni 2017). Invasive species (i.e., aliens that become detrimental to native species and communities) often possess traits that confer a competitive advantage, such as producing and releasing allelopathic substances, being more aggressive, growing larger, having a more efficient reproductive strategy, or being poisonous or inedible to local predators (Callaway and Ridenour 2004). While invaders may sometimes possess novel weapons or defenses, native species may evolve responses to mitigate the costs of counteracting them.

Coevolution, an ecologically driven evolutionary process between two or more species, is widespread and can occur rapidly (Thompson 1994). Biological invasions are significant drivers of rapid coevolution (Strauss et al. 2006; Moran and Alexander 2014). Native species adapt their morphological, physiological, and behavioural traits within a few generations to lessen the impacts of alien species (Mooney and Cleland 2001). These traits may already exist in the native population but could be rare, unexpressed, or may emerge *de novo* through mutations. Alien species serve as strong selective agents, although time is necessary to increase the frequency of novel traits in the populations of interacting species (Leger and Espeland 2010).

The evolution of defence mechanisms initiates an “arms race”, as alien species are also expected to adapt to simultaneous changes. Populations of aliens and natives differ in trait frequency and the duration of coexistence. Even when coevolution occurs independently across an invasion range, it may vary locally. Consequently, each population may interact uniquely due to distinct environmental selection pressures and local community composition (Thompson 1994, 2010). This variation can create a geographic mosaic of coevolutionary interactions (Thompson 1994), shaping the impacts and community resilience of alien species (Strauss et al. 2006). For example, wild parsnip *Pastinaca sativa* and parsnip webworms *Depressaria pastinacella*, introduced to North America, have coevolved regionally, with plants developing chemical defences and insects evolving detoxification enzymes (Berenbaum et al. 1998; Zangerl and Berenbaum 2003). Similarly, the cardiotoxins produced by the cane toad *Rhinella marina* during its invasion in Australia, which were novel to the Australian fauna, posed a threat to evolutionarily naïve predators such as the Australian black snake *Pseudodechis porphyriacus*. Within 60 years, the snakes evolved greater toxin tolerance, learned to avoid toads as prey, and developed a smaller head and mouth gape, which reduced the consumption of large toxic prey (Phillips and Shine 2006, 2005). Toxin resistance correlated with the time since the invasion front passed. At the same time, invading cane toads have also evolved and are now smaller and less toxic (Phillips and Shine 2005).

We investigated the geographic pattern of rapid coevolution using an unusual host–parasite system involving a parasitic fish, the bitterling, and unionid mussels. Bitterling (family Acheilognathidae) are a species‐rich group that lay their eggs in the gills of freshwater mussels. Most bitterling species are distributed in East Asia and one species, the European bitterling ( *Rhodeus amarus* ), is widely distributed throughout continental Europe. It comprises two major clades (Danubian and Baltic), which colonised Europe following glaciation, with few locally endemic lineages (species) in the southwestern Palearctic (Bartáková et al. 2019). All female bitterling deposit their eggs using a long ovipositor inserted through the mussel's exhalant siphon. The eggs are fertilised when the male releases sperm over the inhalant siphon, which is drawn into the gill cavity (Smith et al. 2004). Before oviposition, bitterling perform conspicuous pre‐spawning behaviours, including male and female inspection of mussel quality (based on oxygen levels and the presence of embryos), the male leading females towards particular mussels, and females skimming over the mussel's siphon (Smith et al. 2002, 2004). These behaviours are good indicators of bitterling preference for specific host mussels (Mills and Reynolds 2002a). Bitterling eggs hatch within 2 days, and embryos attach to the mussel's gills, developing for about a month (Aldridge 1999). They compete with host mussels for oxygen and reduce water circulation through the gills, negatively impacting mussel condition and fecundity (Smith et al. 2001; Mills et al. 2005; Reichard et al. 2006; Labecka and Reichard 2025; Yi et al. 2024). Mussels can defend themselves by ejecting bitterling eggs and embryos, with the ability to do so being higher among Asian than European mussels (Reichard, Liu, and Smith 2007). The European bitterling is a host‐use generalist (Smith et al. 2004; Halabowski et al. 2024) and can readily exploit exotic mussel species (Holčík and Wit 1962; U.S. Fish and Wildlife Service 2019, Pfeiffer et al. 2025) but exhibits oviposition preferences towards particular mussel species and individuals within species (Mills and Reynolds 2002a, 2004).

The Chinese pond mussel *Sinanodonta woodiana* was introduced to Europe from East Asia in the 1970s and has rapidly spread throughout continental Europe in the 21st century (Konečný et al. 2018). *Sinanodonta woodiana* has a long coevolutionary history with several species of East Asian bitterlings (Reichard, Liu, and Smith 2007; Rouchet et al. 2017) and is highly effective at ejecting *R. amarus* eggs (Reichard, Przybylski, et al. 2007). The widespread invasion of *S. woodiana* in Europe has created a spatiotemporal mosaic of interactions with native European bitterling in invaded communities, providing an opportunity to study rapid coevolution in this host–parasite system (Anil et al. 2024). In Poland, *S. woodiana* has been present in some reservoirs for at least 40 years (Zdanowski 1996), while other sites have been invaded over the past few years (Mehler et al. 2024). With the low dispersal ability and strong genetic structuring of European bitterling (Bartáková et al. 2018), the system is ideal for testing hypotheses regarding the coevolutionary mosaic of biological invasions.

This study examines the role of coevolution in the behavioural selection of hosts by comparing the preferences of individual fish for alien mussel hosts across three fish populations, which differ in their sympatry with local parasitic fish. Our methodology accounts for individual mussel quality (which influences choice among native host individuals) and the general geographic context (to compare the effects of alien and native host species with recent (5 years), intermediate (13 years), and longstanding (40+ years) associations).

**FIGURE 1:**
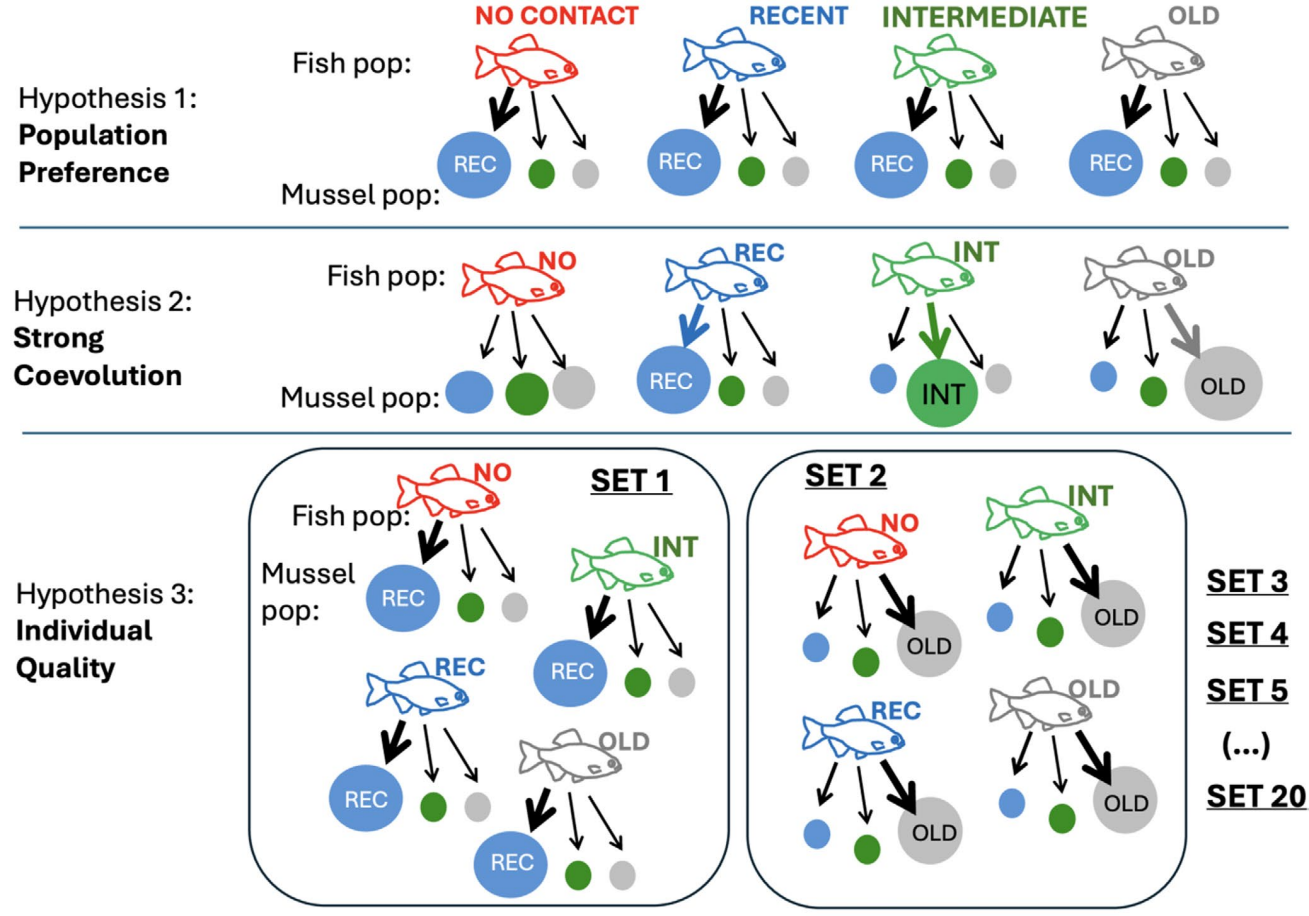
The outline of hypotheses 1–3 showing how prediction on host preference (arrow, with a thicker line denoting significant preference) is distributed across fish and mussel populations for each hypothesis. The colour highlights history of association with recent (blue), intermediate (green), old (grey) and none (red) coexistence between local bitterling and mussel populations.

We specifically tested the role of rapid local coevolution in bitterling host choice by evaluating three competing hypotheses (outlined in Figure 1). First, we hypothesised that the bitterling's choice of host mussel individuals is congruent at the population level, with all bitterling populations consistently basing their decisions on population identity, thus demonstrating preference or avoidance of a specific mussel population (Population Preference Hypothesis). We predicted that bitterling would be able to estimate host suitability and would avoid mussels from the recently established *S. woodiana* population due to the strong negative impacts of recent invaders and a priori knowledge that *S. woodiana* could eject all bitterling eggs at the onset of its invasion (Reichard, Przybylski, et al. 2007). Second, we hypothesised strong coevolution between the partners, which would lead to a preference in bitterling for *S. woodiana* populations with which they coexist locally (sympatric) and to which they have recently adapted through coevolution (Strong Coevolution Hypothesis). Third, we hypothesised that bitterlings base their decisions on the quality of individual mussels, an attribute distinct from population identity (i.e., individual condition) (Individual Quality Hypothesis). Under this hypothesis, we predicted that preferences for individual mussels would be consistent across bitterling populations without any bias towards a specific mussel population. To ascertain whether the pattern of alien host preference depends on rapid, recent coevolution or relates to a much longer coevolutionary history, we complemented our experimental work with alien *S. woodiana* populations by conducting matching experiments using the native mussel host species, *Anodonta anatina* , employing the same experimental design, protocol, and identical field sites as source populations for all mussels and fish. Based on previous studies (Smith et al. 2001; Mills and Reynolds 2002b), we predicted that the bitterling's preference for native *A. anatina* would align with the Individual Quality Hypothesis.

## 2 Methods

### 2.1 *Rhodeus amarus* — *Sinanodonta woodiana* Interaction

European bitterling utilise all native European host mussel species (Halabowski et al. 2024). *Sinanodonta woodiana* has become dominant in many European unionid communities, frequently displacing native species (Benkő‐Kiss et al. 2013; Douda et al. 2024). *Sinanodonta woodiana* evolved in East Asia, a region characterised by high bitterling diversity and abundance (Smith et al. 2004). *Rhodeus amarus* , being the only bitterling species in Europe, apparently faces low resistance to parasitism from local mussels, particularly in the continental part of their range (Reichard et al. 2010), which were colonised relatively recently from glacial refugia (Bryja et al. 2010). Earlier studies indicated that *S. woodiana* can be either behaviourally avoided (Reichard et al. 2015) or utilised indiscriminately, with the ensuing cost of complete clutch ejection by the host mussel (Reichard et al. 2012). Nevertheless, recent observations in captivity suggested the successful development of *R. amarus* embryos in *S. woodiana* (unpublished data). This finding provided the motivation and rationale for the present study, which is designed to test the potential for rapid evolutionary change under strong selection pressure in mussel communities dominated by *S. woodiana* .

### 2.2 Study Sites

The study sites were selected based on previously reported occurrences of bitterling and freshwater mussels, along with a pilot study conducted a year earlier to confirm the presence of *R. amarus* , *S. woodiana* , and *A. anatina* (widespread and locally common European unionid) at these locations. To establish the timeline for the first records of *S. woodiana* at specific sites, we utilised an extensive database published by Mehler et al. (Mehler et al. 2024), supplemented by personal communication with A. M. Łabęcka, the originator of the Mehler et al. database. We identified four study sites according to the presence of *S. woodiana* : (1) a site without *S. woodiana* —the source of *R. amarus* naïve to *S. woodiana* (Drzewiczka River; N 51.45037, E 20.48654); (2) a site with recent sympatry ( *S. woodiana* first recorded in 2018, i.e., 5 years prior to this study) (Pilica River; N 51.833734, E 21.270223); (3) a site with intermediate sympatry ( *S. woodiana* first recorded in 2010) (Krajskie Oxbow Lake; N 50.012960, E 19.530882); and (4) a site where *S. woodiana* has coexisted with the local *R. amarus* population for the longest duration (old sympatry group, with *S. woodiana* first recorded in the early 1980s—the first recorded locality of this species in Poland, i.e., approximately 40 years ago) (Licheńskie Lake; N 52.312995, E 18.349566).

Fish were collected by electrofishing (EFGI 650, BSE Bretschneider Spezialelektronik, Chemnitz, Germany) and transported to the laboratory in aerated containers. Mussels were collected by hand by searching the sediment.

### 2.3 Experimental Setup

Behavioural experiments were conducted in the aquarium facility at the University of Lodz, Poland, from 3 May to 27 June 2023. Prior to the experiment, fish and mussels were kept in large outdoor tubs (130 × 130 cm, 1 m deep) in the University botanic garden. Each morning, female bitterling were checked for an extended ovipositor, an unambiguous indication of ovulation. Fish that were ready to spawn were collected and transferred to the aquarium facility. Haphazardly selected males in breeding colouration and reproductively active female bitterlings were placed in an aquarium (60 × 40 cm, 50 cm deep) containing three *S. woodiana* mussels (one individual from each experimental population). Initially, the females were isolated in a transparent container to encourage male inspection of the host mussels. After a 40‐min acclimation period, the females were released, and preferences of both males and females for specific mussels were observed over a 30‐min period following the first signs of reproductive behaviour.

Mussel‐directed preference behaviours were used to quantify these preferences. Bitterling males led females to specific mussels and ejaculated over preferred mussels as part of courtship. Females inspected mussels prior to spawning and displayed conspicuous “skimming” behaviour over chosen mussels before egg laying. Reproductive behaviour was recorded for 20 pairs of bitterling from each of four bitterling populations (3 sympatric with *S. woodiana* : recent, intermediate, and old; and one naïve to *S. woodiana* , without any contact with *S. woodiana* ). Each pair of fish was tested for their choice among mussels from three mussel populations (old, intermediate, recent) presented simultaneously. The mussel sets were kept in groups and successively offered to fish pairs from all four populations to enable testing of hypothesis #3 (Mussel Quality) on individual congruence arising from variation in individual mussel quality (irrespective of population identity). This design gave a total of 20 *S. woodiana* sets and 20 bitterling pairs from each of the four populations tested.

An identical approach was employed for sets of *A. anatina* mussels. This mussel species is the closest relative to *S. woodiana* among native European freshwater mussels and was previously classified within the same genus due to its similar morphology. We also selected *A. anatina* mussels with shell sizes comparable to those of *S. woodiana* . It is important to note that the Population Preference Hypothesis *for* *A. anatina* (unlike in *S. woodiana* ) was not associated with the duration of sympatry (which was long for all population combinations) but could indicate a potential preference or avoidance of a specific *A. anatina* population.

### 2.4 Data Analysis

All data were inspected for errors, outliers, and missing values. We used the behavioural preference as the most effective approach to measure mussel preference. In males, we employed the ejaculation rate, which serves as a clear indicator of investment in a specific mussel prior to oviposition in *R. amarus* (Smith et al. 2014). Males sweep forward over the inhalant siphon of the mussel during ejaculation, typically performing this 1–8 times before female spawning. In females, the skimming rate was used to assess mussel preference. Mussel skimming resembles spawning, although the ovipositor is not inserted into the mussel. The function of skimming remains unclear (whether it tests mussel response or attracts a partner; Smith et al. 2004), but it clearly indicates a preference for a particular mussel individual (Mills and Reynolds 2002b). Spawning decisions were also recorded, but these were relatively rare during the 30‐min observation period.

Generalised Linear Models were employed to test Hypothesis #1 (Population Preference) and Hypothesis #2 (Strong Coevolution), with population identity (three levels) or sympatry (two levels: Sympatric, Allopatric) as the primary factors. We were also interested in fish population‐specific responses and modelled fish population identity (four levels) along with its interactions with mussel population identity. A significant interaction term could indicate whether fish population history influenced their behaviour towards a particular mussel population. For the Population Preference Hypothesis, we constructed three models for each dataset, which included (1) mussel population origin, fish population origin, and their interaction, (2) mussel population origin and fish population origin, and (3) only mussel population origin. We employed a similar approach to test the Strong Coevolution Hypothesis, substituting the “mussel origin” factor with “sympatry”. In modelling the role of sympatry, we excluded bitterling populations without natural contact with *S. woodiana*, as all three test mussels were allopatric to them. Fish size and mussel size were included as covariates in the initial models but had no significant effect and were subsequently omitted from the final models. Replicate ID served as a random intercept to group data from a fish pair exposed to the set of three mussels (the equivalent of a “paired design”). We utilised a Tweedie error distribution, which is appropriate for zero‐inflated data (Bonat et al. 2018). We assessed zero inflation, over‐ and under‐dispersion of residuals, and model misspecification using the *DHARMa* package (Hartig 2021). To test the Mussel Quality Hypothesis, we analysed the consistency of preference for particular mussel individuals within each set of three mussels across four bitterling pairs (one for each bitterling population) using Kendall concordance coefficients (*W*).

The original data and script for data analysis are available on the Figshare repository (https://doi.org/10.6084/m9.figshare.28644491).

## 3 Results

### 3.1 Bitterling Preferences for Alien Mussels ( *Sinanodonta woodiana* )

#### 3.1.1 Population Preference Hypothesis

**FIGURE 2:**
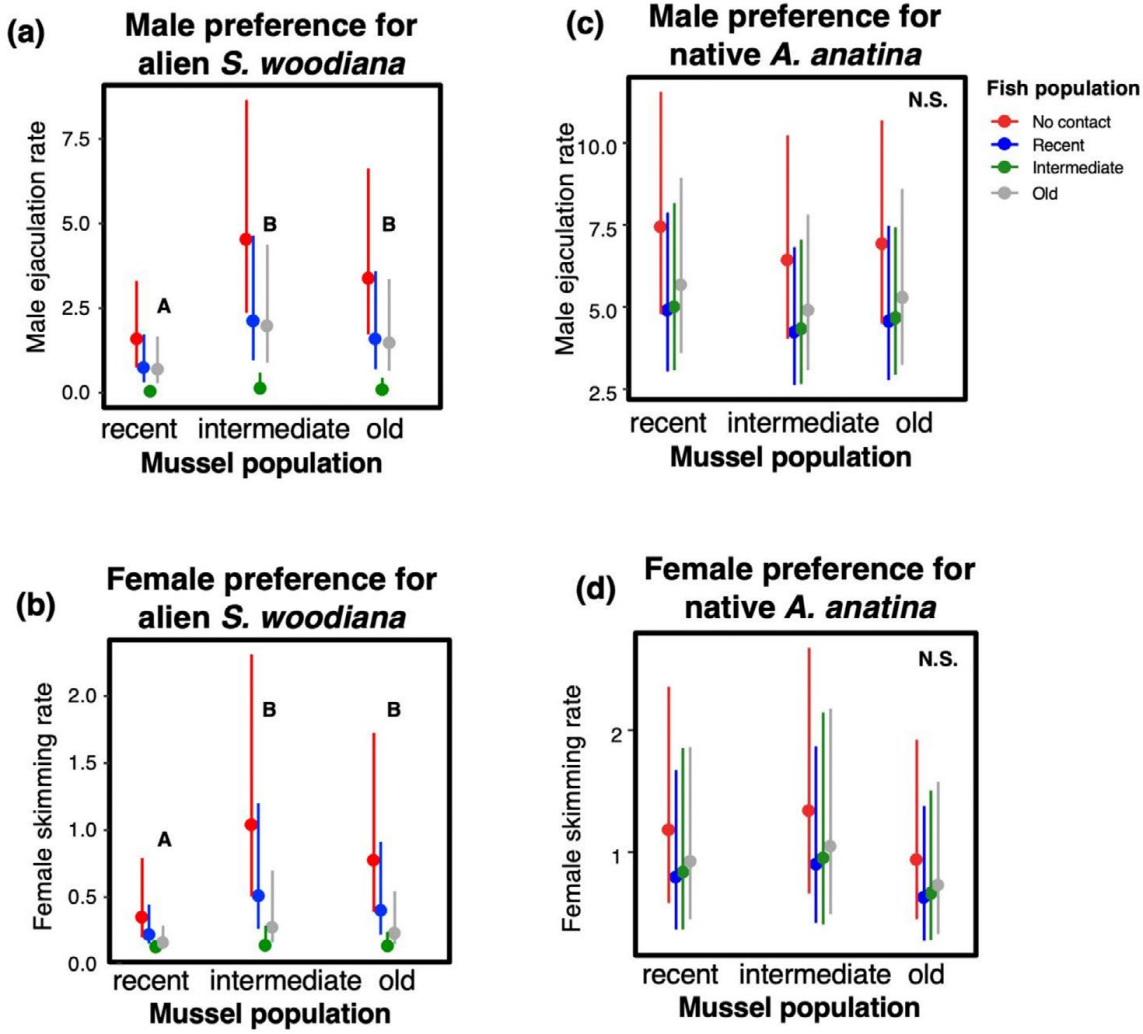
The rates of male ejaculation (a, c) and female skimming (b, d) over the siphons of mussels of particular origin by fish from populations without (red), recent (blue), intermediate (green) and old (grey) coexistence with local bitterling population with *Sinanodonta woodiana* (a, b) and *Anodonta anatina* (c, d). Means and one standard error from model estimates are shown. Letters denote statistically different subgroups (*p* < 0.05).

Bitterling response to the alien mussel species aligned with predictions from the Population Preference Hypothesis, as bitterling consistently avoided mussels from the recently established *S. woodiana* population (Figure 2a). The best models for both males and females included mussel and fish origins but not their interactions, supporting the absence of population‐specific responses. In males, the ejaculation rate was significantly lower over mussels from the recently established population (Tweedie GLMM: the contrast between recent and intermediate: *z* = 3.21, *p* = 0.001, the contrast between recent and old: *z* = 2.25, *p* = 0.025). While fish populations significantly varied in their ejaculation responses (Table 1a; *χ* ^2^ = 30.58, d.f. = 3, *p* < 0.001), they were congruent in their preferences for *S. woodiana* populations (Figure 2a), as the model incorporating the interaction between mussel and fish origins did not yield a significantly superior fit to the data (*χ* ^2^ = 7.53, d.f. = 6, *p* = 0.274). In females, the skimming rate was also lowest over mussels from the recently established *S. woodiana* population (Tweedie GLMM: the contrast between recent and intermediate: *z* = 2.98, *p* = 0.003, the contrast between recent and old: *z* = 2.16, *p* = 0.031). Similar to males, fish populations varied in their overall skimming rate (Table 1b; *χ* ^2^ = 21.31, d.f. = 3, *p* < 0.001), but their preferences were congruent (Figure 2b), and the model with the interaction between mussel and fish populations provided a poorer fit to the data (*χ* ^2^ = 3.12, d.f. = 6, *p* = 0.793).

**TABLE 1:** Results of generalised linear mixed models with Tweedie distribution on (a) male ejaculation frequency and (b) female skimming frequency to test the Population Preference Hypothesis in *Sinanodonta woodiana* . Estimated coefficients (and their 95% confidence interval and statistical significance of their contrast with reference treatment: Mussel with recent association, fish without association) for a combination of *S. woodiana* mussel origin treatment (recent, intermediate, old association with bitterling) and bitterling origin treatment (fish from populations without, with recent, intermediate, and old association with *S. woodiana* mussels).

Coefficient	(a) Male ejaculation			(b) Female skimming		
	Estimates	Conf. Int.	*p*	Estimates	Conf. Int	*p*
Intercept (recent)	0.46	−0.28 to 1.20	0.221	−1.49	−2.58 – −0.40	0.007
Mussel (intermediate)	1.05	0.41–1.69	0.001	1.40	0.48–2.33	0.003
Mussel (old)	0.76	0.10–1.42	0.025	1.06	0.10–2.02	0.031
Fish (recent)	−0.76	−1.63 to 0.12	0.090	−0.86	−1.96 – 0.24	0.124
Fish (intermediate)	−3.51	−5.00 to −2.02	< 0.001	−4.11	−6.53 – −1.69	0.001
Fish (old)	−0.83	−1.71 to 0.06	0.066	−1.83	−3.17 to −0.48	0.008

Random effects		
*σ* ^2^ (*τ*_00 replicateID_)	1.66 (0.86)	2.54 (1.34)
Marg *R* ^2^/Cond *R* ^2^	0.440/0.631	0.412/0.615
*N*_obs_ (*N*_replicateID_)	240 (80)	240 (79)

#### 3.1.2 Strong Coevolution Hypothesis

**FIGURE 3:**
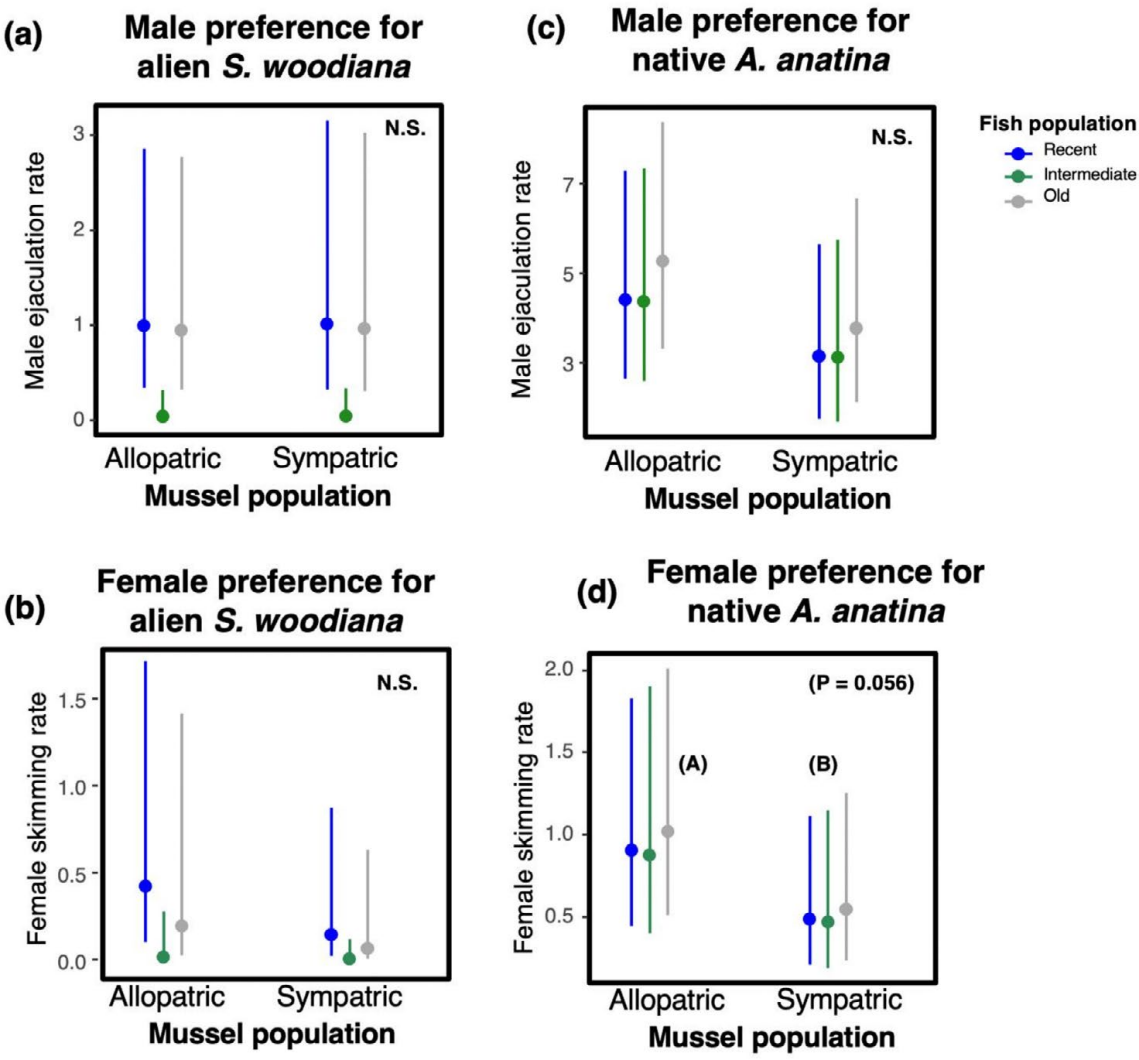
The rates of male ejaculation (a, c) and female skimming (b, d) over the siphons of mussels sympatric or allopatric with bitterling populations. The colour highlights fish population identity for recent (blue), intermediate (green) and old (grey) coexistence with the local bitterling population. Results for *Sinanodonta woodiana* (a, b) and *Anodonta anatina* (c, d) are shown. Means and one standard error from model estimates are shown. Letters denote statistically different subgroups (*p* < 0.05).

We found no evidence to support a preference for mussels from sympatric *S. woodiana* populations by either male (Tweedie GLMM on ejaculation: *χ* ^2^ = 0.01, d.f. = 1, *p* = 0.954; Table 2a, Figure 3a) or female bitterling (Tweedie GLMM on skimming: *χ* ^2^ = 2.16, d.f. = 1, *p* = 0.142; Table 2b, Figure 3b). Overall, the behaviour of bitterling populations varied regarding their interaction with mussels (males: *χ* ^2^ = 15.25, d.f. = 2, *p* = 0.001; females: *χ* ^2^ = 8.06, d.f. = 2, *p* = 0.018) but not in a population‐specific manner (interactions, males: *χ* ^2^ = 0.30, d.f. = 2, *p* = 0.862; females: *χ* ^2^ = 0.32, d.f. = 2, *p* = 0.850).

**TABLE 2:** Results of generalised linear mixed models with Tweedie distribution on (a) male ejaculation frequency and (b) female skimming frequency to test the Strong Coevolution Hypothesis in *Sinanodonta woodiana* . Estimated coefficients (and their 95% confidence interval and statistical significance of their contrast with reference treatment: Allopatric relationship with recent fish association population) for a combination of *S. woodiana* mussel sympatry (allopatric or sympatric with specific bitterling population) and bitterling origin treatment (fish from populations with recent, intermediate, and old association with *S. woodiana* mussels).

Coefficient	(a) Male ejaculation			(b) Female skimming		
	Estimates	Conf. Int.	*p*	Estimates	Conf. Int	*p*
Intercept (allopatric)	−0.01	−1.06 to 1.05	0.992	−0.86	−2.27 to 0.54	0.229
Sympatry (sympatric)	0.02	−0.67 to 0.70	0.959	−1.07	−2.59 to 0.44	0.165
Fish (intermediate)	−3.14	−5.07 to −1.21	0.001	−3.27	−6.05 to −0.50	0.021
Fish (old)	−0.05	−1.29 to 1.20	0.942	−0.77	−2.24 to 0.71	0.308

*Random effects*		
*σ* ^2^ (*τ*_00 replicateID_)	2.24 (2.30)	3.27 (0.71)
Marg *R* ^2^/Cond *R* ^2^	0.323/0.666	0.361/0.475
*N*_obs_ (*N*_replicateID_)	180 (60)	180 (59)

#### 3.1.3 Mussel Quality Hypothesis

There was no support for the Mussel Quality Hypothesis regarding the alien *S. woodiana* , as individual mussel preferences were not congruent among fish from different bitterling populations (Kendall chi‐squared test, males: Wt = 0.196, *χ* ^2^ = 184.4, d.f. = 4, 236, *p* = 0.994; females: Wt = 0.252, *χ* ^2^ = 237.1, d.f. = 4, 236, *p* = 0.450).

### 3.2 Bitterling Preference for Native Mussels ( *Anodonta anatina* )

#### 3.2.1 Population Preference Hypothesis

Bitterling response to the native mussel species did not align with the predictions set out by the Population Preference Hypothesis. Bitterling exhibited neither a preference for nor avoidance of any specific *A. anatina* population (Figure 2c,d) (Tweedie GLMM, mussel origin; males: χ^2^ = 0.49, d.f. = 2, *p* = 0.783, Table 3a; females: *χ* ^2^ = 1.23, d.f. = 2, *p* = 0.542, Table 3b). Furthermore, fish population origin did not influence the interaction with *A. anatina* mussels overall (males: *χ* ^2^ = 3.06, d.f. = 3, *p* = 0.382; females: *χ* ^2^ = 0.99, d.f. = 3, *p* = 0.803), nor did it affect interactions with specific *A. anatina* populations (males: *χ* ^2^ = 12.84, d.f. = 6, *p* = 0.052; females: *χ* ^2^ = 10.37, d.f. = 6, *p* = 0.110). The population‐specific estimates from the full additive model are presented in Appendix S1: Table S1.

**TABLE 3:** Results of generalised linear mixed models with Tweedie distribution on (a) male ejaculation frequency and (b) female skimming frequency to test the Population Preference Hypothesis in *Anodonta anatina* . Estimated coefficients (and their 95% confidence interval and statistical significance of their contrast with reference treatment: Fish with a recent association) for a combination of *A, anatina* mussel origin treatment (sites with a recent, intermediate, old association of *S. woodiana* with bitterling fish).

Coefficient	(a) Male ejaculation			(b) Female skimming		
	Estimates	Conf. Int.	*p*	Estimates	Conf. Int	*p*
Intercept (recent)	1.76	1.43–2.08	< 0.001	−0.03	−0.52 to 0.46	0.913
Mussel (intermediate)	−0.17	−0.64 to 0.30	0.487	0.12	−0.46 to 0.70	0.692
Mussel (old)	−0.06	−0.52 to 0.40	0.791	−0.22	−0.83 to 0.39	0.479

*Random effects*		
*σ* ^2^ (τ_00 replicateID_)	1.19 (0.00)	1.59 (0.54)
Marg *R* ^2^/Cond *R* ^2^	0.004/NA	0.009/0.261
*N*_obs_ (*N*_replicateID_)	240 (80)	240 (80)

#### 3.2.2 Strong Coevolution Hypothesis

The effect of sympatry had no influence on the distribution of male bitterling ejaculations among *A. anatina* mussels (Figure 3c) (*χ* ^2^ = 2.03, d.f. = 1, *p* = 0.154; Table 4a), whereas female bitterling showed a tendency to skim more over allopatric mussels (*χ* ^2^ = 3.64, d.f. = 1, *p* = 0.056; Table 4b, Figure 3d). Regarding the origin of *A. anatina* populations, the fish population of origin had no impact on native host selection in relation to sympatry (males: *χ* ^2^ = 0.46, d.f. = 2, *p* = 0.796; females: *χ* ^2^ = 0.12, d.f. = 2, *p* = 0.943), nor was there an effect from the interaction (males: *χ* ^2^ = 3.92, d.f. = 2, *p* = 0.141; females: *χ* ^2^ = 0.90, d.f. = 2, *p* = 0.639). The population‐specific estimates from the full additive model can be found in Appendix S1: Table S2.

**TABLE 4:** Results of generalised linear mixed models with Tweedie distribution on (a) male ejaculation frequency and (b) female skimming frequency to test the Strong Coevolution Hypothesis in *Anodonta anatina* . Estimated coefficients (and their 95% confidence interval and statistical significance of the contrast between allopatric or sympatric association with particular *A. anatina* population).

Coefficient	(a) Male ejaculation			(b) Female skimming		
	Estimates	Conf. Int.	*p*	Estimates	Conf. Int	*p*
Intercept (allopatric)	1.55	1.21–1.89	< 0.001	−0.06	−0.55 – 0.42	0.802
Sympatry (sympatric)	−0.34	−0.81 – 0.13	0.155	−0.62	−1.26 – 0.02	0.059

*Random effects*		
*σ* ^2^ (*τ*_00 replicateID_)	1.15 (0.28)	1.61 (0.78)
Marg *R* ^2^/Cond *R* ^2^	0.018/0.208	0.035/0.350
*N*_obs_ (*N*_replicateID_)	180 (60)	180 (60)

#### 3.2.3 Mussel Quality Hypothesis

As predicted, native mussels were selected by bitterling based on their individual quality. A significant effect of congruent mussel preference was observed in females (Kendall chi‐squared test, Wt = 0.281, *χ* ^2^ = 278.7, d.f. = 4, 229, *p* = 0.012), while males displayed a non‐significant tendency (Wt = 0.306, *χ* ^2^ = 254.9, d.f. = 4, 228, *p* = 0.099).

### 3.3 Oviposition

Overall, bitterling of both sexes responded to native *A. anatina* approximately four times more frequently compared to alien *S. woodiana* (compare left and right panels in Figures 2 and 3). There were 28 ovipositions (in 22 replicates, i.e., 9.2% of replicates) in invasive *S. woodiana* , which was significantly less than 79 ovipositions (in 54 replicates, 21.7%) in native *A. anatina* across a total of 80 observations for each mussel species (Fisher's exact test, *p* < 0.001). The distribution of ovipositions corresponded with behavioural preferences but lacked statistical power. There was no significant difference in oviposition in *S. woodiana* from different populations (eggs laid to 4, 9, and 9 mussels from recent, intermediate, and old associations, respectively; Fisher's exact test, *p* = 0.326) or in *A. anatina* (22, 12, and 18 ovipositions from different populations; Fisher's exact test, *p* = 0.182). No significant effect of sympatry was found for *S. woodiana* (oviposition in sympatric mussels: 3.3%, allopatric 11.2%; Fisher's exact test, *p* = 0.075) nor for *A. anatina* (sympatric: 18.3%, allopatric 23.9%; Fisher's exact test, *p* = 0.476).

## 4 Discussion

We studied the role of coevolution in the behavioural choices made by a native parasitic fish regarding a novel host, utilising an experimental approach within a comparative framework. By combining three alien host mussel populations with four populations of native bitterling fish, we demonstrate that alien mussels with the most recent (i.e., 5 years) coexistence with the European bitterling were avoided by all bitterling populations, including the population that was naïve to alien mussels. We found no effect of sympatry or individual host quality on the choice of alien hosts by bitterling. This finding supports the Population Preference Hypothesis for interactions between European bitterling and alien host species, as opposed to the Strong Coevolution Hypothesis or the Individual Quality Hypothesis. In contrast, different pairs of bitterling consistently preferred specific individuals of the native mussel species, regardless of their population origin, affirming previous evidence for the Individual Quality Hypothesis defined for interactions between European bitterling and native hosts (Smith et al. 2001; Mills and Reynolds 2002b). This finding indicates that bitterling utilise different cues when assessing native and alien hosts. Bitterling populations varied in the intensity of their reproductive behaviours in response to alien hosts but maintained consistency in their overall preferences. Overall, bitterling clearly expressed more interest towards native than alien mussel species.

### 4.1 Alien Species Perspective

All European bitterling populations consistently avoided the alien host population with the most recent sympatry. This observation supports a prior conclusion that European bitterling readily utilize certain *S. woodiana* populations for oviposition while avoiding others (Reichard et al. 2015). In previous tests, European bitterling were able to distinguish between three unionid mussel species: two native *Anodonta* species ( *A. anatina* and *A. cygnea* ) and the alien *S. woodiana* . In that study, European bitterling did not avoid (and instead readily utilized) *S. woodiana* for oviposition from a site with sympatry (Lake Licheńskie), despite this being followed by the rejection of all bitterling eggs during the initial days of development (Reichard et al. 2015). In contrast, European bitterling entirely avoided ovipositing into mussels from another *S. woodiana* population established in Czechia (Danubian region), even when it was presented as the sole host for oviposition. Although this particular population was not included in the design of the current study, it was originally tested during the first 10 years of its local coexistence with European bitterling (Reichard et al. 2012), analogous to the coexistence period of the recent association population of *S. woodiana* . The avoidance of oviposition in that recently sympatric *S. woodiana* population protected bitterling from the adverse effects of egg ejection by *S. woodiana* (Reichard et al. 2015). Studies on other bitterling and mussel species in East Asia have similarly documented significant population‐level variation in host preference and performance (Rouchet et al. 2017; Kitamura et al. 2012), although the source of these differences has not yet been identified.

The recently established *S. woodiana* population can be likened to an invasion front. The invasion front represents the advancing edge of the alien species' range in the new region and includes individuals with specific traits that favour invasion (Phillips et al. 2006) and are often simultaneously more harmful to local species (Simberloff et al. 2013). The evolutionary history of *S. woodiana* with East Asian bitterlings likely provides it with an advantage in recognising and rejecting parasitic bitterling eggs. While we studied mussel choice behaviour until oviposition, previous field and mesocosm research indicated that *Rhodeus amarus* embryos are forcefully ejected by *S. woodiana* during development (Reichard et al. 2015; Marčić et al. 2024). Our recent observations suggested successful embryo development in *S. woodiana* (which motivated the present study), potentially driven by rapid evolutionary changes under strong selection pressure in mussel communities dominated by *S. woodiana* (unpublished data). Unfortunately, recording the survival of bitterling eggs in host mussels was not compatible with our study design due to the repeated use of the same mussel set.

In contrast, the European bitterling has coevolved with *A. anatina* and other European unionids, which result in greater compatibility and bitterling reproductive success (Mills and Reynolds 2002a). The bitterling preference for host mussels of native *A. anatina* matched the predicted selection scenario for the most suitable individual mussel (Mills and Reynolds 2002a; Smith et al. 2001), irrespective of mussel population origin and congruently among bitterling pairs and populations.

### 4.2 Native Species Perspective

From the perspective of inter‐population variation among native species, the population of bitterling naïve to *S. woodiana* showed the highest interest in the alien host. The absence of prior evolutionary coexistence may have resulted in an inability to assess host quality and suggests that previous experience with unsuitable alien hosts prompts behavioural avoidance. Indeed, the behaviour of native species is crucial in interactions with alien species and often results in their avoidance across ecological interactions such as pollination, parasitism, or predation (Traveset and Richardson 2006; Lymbery et al. 2014; Saul and Jeschke 2015). For instance, native bison ( *Bos bison* ) and elk ( *Cervus elaphus* ) avoid foraging in areas dominated by alien *Euphorbia esula* plants that produce toxic latex (Bourchier et al. 2006; Keane and Crawley 2002). The efficiency of native predators is diminished due to habitat modifications caused by alien species, leading to the avoidance of such areas (Stewart et al. 2021), while the naiveté of native prey renders them more vulnerable to alien predators (Cox and Lima 2006).

Our results demonstrated that the initial vigilance or inability to utilize novel hosts has not vanished in the bitterling fish over at least 40 years since the introduction of *S. woodiana* . Given that the reproductive success of European bitterling ovipositing in *S. woodiana* is significantly compromised (Reichard et al. 2012), avoiding unknown or unsuitable hosts emerges as an effective strategy to minimize reproductive loss in rapidly changing environments. Suggested evidence for successful embryo development in *S. woodiana* under particular conditions (unpublished data) marks a notable departure from earlier conclusions that *S. woodiana* entirely impedes *R. amarus* reproduction (Reichard, Przybylski, et al. 2007). This situation may reflect rapid evolutionary changes driven by strong selection pressures in mussel communities dominated by *S. woodiana* .

We also observe that male and female host preferences were congruent. This finding aligns with previous observations indicating that the intensity of female reproductive behaviour (recorded as the skimming rate) increases with a higher rate of male sperm release (Smith et al. 2004). However, such consistency is not always a rule. Spence et al. (2013) demonstrated that males exhibited increased ejaculation rates in fertilisation in the presence of a new mussel, while females were more selective in choosing mussels regarding their suitability for offspring survival.

### 4.3 Native‐Alien Species Interactions

Behavioural mechanisms that stabilise the coexistence of native and alien species have been reported from other ecological relationships, wherein alien species become less detrimental following the evolution of behavioural or physiological adaptations in native partners (Phillips and Shine 2006). This outcome is most prevalent in invader‐dominated communities, characteristic of long‐term invasions, where the selection pressure is consistent and strongest (Strayer et al. 2006).

The geographic variability of *R. amarus* responses to *S. woodiana* (as demonstrated by the differing responses of *R. amarus* populations compared to *S. woodiana* populations) also aligns with the geographic mosaic theory of coevolution (Thompson 1994). In regions where *S. woodiana* dominates mussel communities, bitterlings may experience stronger selective pressures, favouring individuals with traits that enhance compatibility with this invasive host. Conversely, in areas with lower densities of *S. woodiana* , the evolutionary pressure to adapt may be less pronounced, resulting in a persistent preference for native mussels. The presence of native mussel hosts such as *A. anatina* , *Unio pictorum* , and *U. tumidus* for oviposition at all our collection sites (Halabowski et al. 2025) likely explains the absence of adjustments and evolution in behavioural and oviposition choices in bitterlings. It remains to be tested whether selection pressures may favour bitterling individuals with traits that enhance compatibility with this invasive host in regions where *S. woodiana* strongly dominates unionid mussel communities (Paunović et al. 2006; Benkő‐Kiss et al. 2013). Conversely, in areas where native mussels remain prevalent, conventional host preferences are likely to persist. While a higher number of fish and mussel populations with replicated patterns of temporal and spatial coexistence are needed for a powerful test of coevolutionary hotspots and coldspots, our study highlights the importance of spatial heterogeneity in shaping evolutionary trajectories at a fine scale.

Chinese pond mussels experience lower parasite pressure compared to sympatric native unionid mussels (Deng et al. 2024), including parasitism by the bitterling, as demonstrated in the current study. The reduced prevalence and abundance of parasites are one of the competitive advantages that alien species gain in their new range (Torchin et al. 2001; Keane and Crawley 2002; Colautti et al. 2004), a phenomenon known as the enemy release hypothesis (Torchin et al. 2003). This reduction in parasitism can facilitate their establishment and spread. Non‐native animal populations harbour fewer parasite species compared to sympatric native species (e.g., Cuban treefrog *Osteopilus septentrionalis* ; Roznik et al. 2020, amphipod *Gammarus roeseli*; Bevins 2019), as well as a less diverse parasite community than populations in their native range (e.g., round goby *Neogobius melanostomus* ; Gendron et al. 2011). Similarly to other species, the lower parasite pressure observed in *S. woodiana* (Deng et al. 2024; Halabowski et al. 2025) may have provided a competitive advantage over native unionid mussels and contributed to the establishment and expansion of *S. woodiana* in novel environments.

The interaction between *R. amarus* and *S. woodiana* serves as an example for understanding the broader implications of biological invasions and associated rapid coevolution (Reichard, Liu, and Smith 2007; Reichard et al. 2012; Reichard, Przybylski, et al. 2007; Shine 2012; Stuart et al. 2014). Coevolutionary dynamics are considered to modulate impacts in invasion biology but may present a significant source of variation in outcomes (Taraschewski 2006; Reichard et al. 2015). The impacts of invasive species change over time (Phillips and Shine 2005), and ecological and evolutionary processes such as learning have been identified as key drivers of this variation (Strayer et al. 2006; Laland 2015). Such evolutionary changes have been documented in invasive populations, leading to a reduced impact on native species (Lankau et al. 2009). We predict that the capacity of *R. amarus* to adjust its behaviour and evolve to exploit *S. woodiana* competently may align with conclusions from other study systems, such as the cane toad invasion in Australia (Phillips and Shine 2006) or the rapid niche shift observed in native green anoles *Anolis carolinensis* following the invasion of *Anolis sagrei* , which exhibited significant changes in ecology and morphology in less than 20 generations (Stuart et al. 2014). We conclude that the lack of evolved use of *S. woodiana* by European bitterling populations results from either sufficient access to suitable native hosts or a failure to evolve traits that facilitate the use of a more challenging host.

## Author Contributions

**Dariusz Halabowski:** conceptualization (equal), data curation (equal), formal analysis (supporting), investigation (lead), methodology (equal), project administration (equal), resources (equal), writing – original draft (equal), writing – review and editing (equal). **Abhishek Nair Anil:** investigation (equal), methodology (supporting), writing – original draft (supporting), writing – review and editing (equal). **Grzegorz Zięba:** investigation (equal), writing – review and editing (equal). **Kacper Pyrzanowski:** investigation (equal), writing – review and editing (equal). **Joanna Grabowska:** investigation (equal), writing – original draft (supporting), writing – review and editing (equal). **Carl Smith:** formal analysis (equal), investigation (equal), writing – review and editing (equal). **Martin Reichard:** conceptualization (lead), data curation (equal), formal analysis (equal), funding acquisition (lead), investigation (supporting), methodology (equal), project administration (equal), supervision (lead), visualization (lead), writing – original draft (equal), writing – review and editing (equal).

## Conflicts of Interest

The authors declare no conflicts of interest.

## Supporting information


**Appendix S1:** Table S1.
**Appendix S1:** Table S2.

## Data Availability

All data generated or analysed during this study was uploaded to the Figshare repository (https://doi.org/10.6084/m9.figshare.28644491).

## References

[ece372270-bib-0001] Aldridge, D. C. 1999. “Development of European Bitterling in the Gills of Freshwater Mussels.” Journal of Fish Biology 54: 138–151.

[ece372270-bib-0002] Anil, A. N. , I. Mehdi , K. Douda , C. Smith , and M. Reichard . 2024. “Reciprocal Transplant Experiments Demonstrate a Dynamic Coevolutionary Relationship Between Parasitic Mussel Larvae and Bitterling Fishes.” Freshwater Biology 69: 1525–1536.

[ece372270-bib-0003] Bartáková, V. , J. Bryja , M. Reichard , V. Bartáková , J. Bryja , and M. Reichard . 2018. “Fine‐Scale Genetic Structure of the European Bitterling at the Intersection of Three Major European Watersheds.” BMC Evolutionary Biology 18: 1–15. 29973160 10.1186/s12862-018-1219-9 PMC6030748

[ece372270-bib-0004] Bartáková, V. , J. Bryja , R. Šanda , et al. 2019. “High Cryptic Diversity of Bitterling Fish in the Southern West Palearctic.” Molecular Phylogenetics and Evolution 133: 1–11. 30586649 10.1016/j.ympev.2018.12.025

[ece372270-bib-0005] Benkő‐Kiss, Á. , Á. Ferincz , N. Kováts , and G. Paulovits . 2013. “Spread and Distribution Pattern of *Sinanodonta woodiana* in Lake Balaton.” Knowledge and Management of Aquatic Ecosystems 408: 9.

[ece372270-bib-0006] Berenbaum, M. R. , A. R. Zangerl , M. R. Berenbaum , and A. R. Zangerl . 1998. “Chemical Phenotype Matching Between a Plant and Its Insect Herbivore.” Proceedings of the National Academy of Sciences of the United States of America 95: 13743–13748. 9811871 10.1073/pnas.95.23.13743 PMC24890

[ece372270-bib-0007] Bevins, S. N. 2019. “Parasitism, Host Behavior, and Invasive Species.” In Encyclopedia of Animal Behavior, edited by J. C. Choe , 2nd ed. Elsevier, Academic Press.

[ece372270-bib-0008] Bonat, W. H. , B. Jørgensen , C. C. Kokonendji , J. Hinde , and C. G. B. Demétrio . 2018. “Extended Poisson–Tweedie: Properties and Regression Models for Count Data.” Statistical Modelling 18: 24–49.

[ece372270-bib-0009] Bourchier, R. , R. Hansen , R. Lym , et al. 2006. Biology and Biological Control of Leafy Spurge. Forest Health Technology Enterprise Team. U.S. Department of Agriculture.

[ece372270-bib-0010] Bryja, J. , C. Smith , A. Konečný , and M. Reichard . 2010. “Range‐Wide Population Genetic Structure of the European Bitterling ( *Rhodeus amarus* ) Based on Microsatellite and Mitochondrial DNA Analysis.” Molecular Ecology 19: 4708–4722. 20958813 10.1111/j.1365-294X.2010.04844.x

[ece372270-bib-0011] Callaway, R. M. , and W. M. Ridenour . 2004. “Novel Weapons: Invasive Success and the Evolution of Increased Competitive Ability.” Frontiers in Ecology and the Environment 2: 436–443.

[ece372270-bib-0012] Colautti, R. I. , A. Ricciardi , I. A. Grigorovich , and H. J. MacIsaac . 2004. “Is Invasion Success Explained by the Enemy Release Hypothesis?” Ecology Letters 7: 721–733.

[ece372270-bib-0013] Cox, J. G. , and S. L. Lima . 2006. “Naiveté and an Aquatic–Terrestrial Dichotomy in the Effects of Introduced Predators.” Trends in Ecology & Evolution 21: 674–680. 16879896 10.1016/j.tree.2006.07.011

[ece372270-bib-0014] Crooks, J. A. 2005. “Lag Times and Exotic Species: The Ecology and Management of Biological Invasions in Slow‐motion1.” Écoscience 12: 316–329.

[ece372270-bib-0015] David, P. , E. Thébault , O. Anneville , P.‐F. Duyck , E. Chapuis , and N. Loeuille . 2017. “Impacts of Invasive Species on Food Webs: A Review of Empirical Data.” Networks of Invasion: A Synthesis of Concepts 56: 1–60.

[ece372270-bib-0016] Deng, B. , N. Riccardi , M. Urbańska , T. J. Marjomäki , W. Andrzejewski , and J. Taskinen . 2024. “Lower Parasite Pressure in Invasive Freshwater Bivalves Than in Sympatric Native Unionidae Mussels in Southern European Lakes.” Biological Invasions 27: 10.

[ece372270-bib-0017] Douda, K. , A. Zieritz , B. Vodáková , et al. 2024. “Review of the Globally Invasive Freshwater Mussels in the Genus *Sinanodonta* Modell, 1945.” Hydrobiologia 852: 1243–1273.

[ece372270-bib-0018] Gallien, L. , and M. Carboni . 2017. “The Community Ecology of Invasive Species: Where Are We and What's Next?” Ecography 40: 335–352.

[ece372270-bib-0019] Gendron, A. D. , D. J. Marcogliese , and M. Thomas . 2011. “Invasive Species Are Less Parasitized Than Native Competitors, but for How Long? The Case of the Round Goby in the Great Lakes‐St. Lawrence Basin.” Biological Invasions 14: 367–384.

[ece372270-bib-0020] Halabowski, D. , K. Pyrzanowski , G. Zięba , et al. 2025. “The Impact of Invasive *Sinanodonta woodiana* (Bivalvia, Unionidae) and Mussel Macroparasites on the Egg Distribution of Parasitic Bitterling Fish in Host Mussels.” Scientific Reports 15: 9417. 40108247 10.1038/s41598-025-93717-8 PMC11923366

[ece372270-bib-0021] Halabowski, D. , M. Reichard , K. Pyrzanowski , et al. 2024. “The Depressed River Mussel *Pseudanodonta complanata* as an Occasional Host for the European Bitterling *Rhodeus amarus* .” Knowledge and Management of Aquatic Ecosystems 425: 3.

[ece372270-bib-0022] Hartig, F. 2021. “DHARMa: Residual Diagnostics for Hierarchical (Multi‐Level / Mixed) Regression Models. 0.4.7.”

[ece372270-bib-0023] Hobbs, R. J. , S. Arico , J. Aronson , et al. 2006. “Novel Ecosystems: Theoretical and Management Aspects of the New Ecological World Order.” Global Ecology and Biogeography 15: 1–7.

[ece372270-bib-0024] Holčík, J. , and J. J. D. D. Wit . 1962. “The Taxonomic Characteristics of Hybrid *Rhodeus* .” Copeia 1962: 777–788.

[ece372270-bib-0025] Hulme, P. E. 2007. “Biological Invasions in Europe: Drivers, Pressures, States, Impacts and Responses.” In Biodiversity Under Threat, edited by R. H. R. M. Hester . University Press.

[ece372270-bib-0026] Keane, R. M. , and M. J. Crawley . 2002. “Exotic Plant Invasions and the Enemy Release Hypothesis.” Trends in Ecology & Evolution 17: 164–170.

[ece372270-bib-0027] Kitamura, J. , N. Nagata , J. Nakajima , and T. Sota . 2012. “Divergence of Ovipositor Length and Egg Shape in a Brood Parasitic Bitterling Fish Through the Use of Different Mussel Hosts.” Journal of Evolutionary Biology 25: 566–573. 22268770 10.1111/j.1420-9101.2011.02453.x

[ece372270-bib-0028] Kolar, C. S. , and D. M. Lodge . 2001. “Progress in Invasion Biology: Predicting Invaders.” Trends in Ecology & Evolution 16: 199–204. 11245943 10.1016/s0169-5347(01)02101-2

[ece372270-bib-0029] Konečný, A. , O. P. Popa , V. Bartáková , et al. 2018. “Modelling the Invasion History of *Sinanodonta woodiana* in Europe: Tracking the Routes of a Sedentary Aquatic Invader With Mobile Parasitic Larvae.” Evolutionary Applications 11: 1975–1989. 30459842 10.1111/eva.12700 PMC6231479

[ece372270-bib-0030] Labecka, A. M. , and M. Reichard . 2025. “The Reproductive Costs of Bitterling Fish and Zebra Mussel Parasitism to a Unionid Mussel.” Aquaculture 595: 741515.

[ece372270-bib-0031] Laland, K. N. 2015. “On Evolutionary Causes and Evolutionary Processes.” Behavioural Processes 117: 97–104. 24932898 10.1016/j.beproc.2014.05.008

[ece372270-bib-0032] Lankau, R. A. , V. Nuzzo , G. Spyreas , and A. S. Davis . 2009. “Evolutionary Limits Ameliorate the Negative Impact of an Invasive Plant.” Proceedings of the National Academy of Sciences of the United States of America 106: 15362–15367. 19706431 10.1073/pnas.0905446106 PMC2730356

[ece372270-bib-0033] Leger, E. A. , and E. K. Espeland . 2010. “Coevolution Between Native and Invasive Plant Competitors: Implications for Invasive Species Management.” Evolutionary Applications 3: 169–178. 25567917 10.1111/j.1752-4571.2009.00105.x PMC3352482

[ece372270-bib-0034] Levine, J. M. , and C. M. D'Antonio . 1999. “Elton Revisited: A Review of Evidence Linking Diversity and Invasibility.” Oikos 87: 15–26.

[ece372270-bib-0035] Lockwood, J. L. , P. Cassey , and T. Blackburn . 2005. “The Role of Propagule Pressure in Explaining Species Invasions.” Trends in Ecology & Evolution 20: 223–228. 16701373 10.1016/j.tree.2005.02.004

[ece372270-bib-0036] Lymbery, A. J. , M. Morine , H. G. Kanani , S. J. Beatty , and D. L. Morgan . 2014. “Co‐Invaders: The Effects of Alien Parasites on Native Hosts.” International Journal for Parasitology: Parasites and Wildlife 3: 171–177. 25180161 10.1016/j.ijppaw.2014.04.002 PMC4145144

[ece372270-bib-0037] Marčić, Z. , P. Prenz , S. Horvatić , et al. 2024. “Is Bitterling ( *Rhodeus amarus* (Bloch, 1782)) Threatened by the Invasive Unionid Species *Sinanodonta woodiana* (Lea, 1834)?” Biological Invasions 26: 3417–3431.

[ece372270-bib-0038] Mehler, K. , A. M. Labecka , I. Sîrbu , N. Y. Flores , R. S. E. W. Leuven , and F. P. L. Collas . 2024. “Recent and Future Distribution of the Alien Chinese Pond Mussel *Sinanodonta woodiana* (Lea, 1834) on the European Continent.” Aquatic Invasions 19: 51–72.

[ece372270-bib-0039] Mills, S. C. , and J. D. Reynolds . 2002a. “Host Species Preferences by Bitterling, *Rhodeus sericeus* , Spawning in Freshwater Mussels and Consequences for Offspring Survival.” Animal Behaviour 63: 1029–1036.

[ece372270-bib-0040] Mills, S. C. , and J. D. Reynolds . 2002b. “Mussel Ventilation Rates as a Proximate Cue for Host Selection by Bitterling, *Rhodeus sericeus* .” Oecologia 131: 473–478. 28547721 10.1007/s00442-002-0895-7

[ece372270-bib-0041] Mills, S. C. , and J. D. Reynolds . 2004. “The Importance of Species Interactions in Conservation: The Endangered European Bitterling *Rhodeus sericeus* and Its Freshwater Mussel Hosts.” Animal Conservation 7: 257–263.

[ece372270-bib-0042] Mills, S. C. , M. I. Taylor , and J. D. Reynolds . 2005. “Benefits and Costs to Mussels From Ejecting Bitterling Embryos: A Test of the Evolutionary Equilibrium Hypothesis.” Animal Behaviour 70: 31–37.

[ece372270-bib-0043] Mooney, H. A. , and E. E. Cleland . 2001. “The Evolutionary Impact of Invasive Species.” Proceedings of the National Academy of Sciences of the United States of America 98: 5446–5451. 11344292 10.1073/pnas.091093398 PMC33232

[ece372270-bib-0044] Moran, E. V. , and J. M. Alexander . 2014. “Evolutionary Responses to Global Change: Lessons From Invasive Species.” Ecology Letters 17: 637–649. 24612028 10.1111/ele.12262

[ece372270-bib-0045] Paunović, M. , B. Csányi , V. Simić , B. Stojanovic , and P. Cakic . 2006. “Distribution of *Anodonta* (*Sinanodonta*) *Woodiana* (Rea, 1834) in Inland Waters of Serbia.” Aquatic Invasions 1: 154–160.

[ece372270-bib-0046] Pearson, D. E. , Y. K. Ortega , Ö. Eren , and J. L. Hierro . 2018. “Community Assembly Theory as a Framework for Biological Invasions.” Trends in Ecology & Evolution 33: 313–325. 29605085 10.1016/j.tree.2018.03.002

[ece372270-bib-0047] Pfeiffer, M. , M. Mildner , C. P. Günter , and M. Leschner . 2025. “The Asian Clam *Corbicula fluminea* , an Accidental Host for the European Bitterling *Rhodeus amarus* .” Knowledge and Management of Aquatic Ecosystems 426: 4.

[ece372270-bib-0048] Phillips, B. L. , G. P. Brown , J. K. Webb , and R. Shine . 2006. “Invasion and the Evolution of Speed in Toads.” Nature 439: 803. 16482148 10.1038/439803a

[ece372270-bib-0049] Phillips, B. L. , and R. Shine . 2005. “The Morphology, and Hence Impact, of an Invasive Species (The Cane Toad, *Bufo marinus* ): Changes With Time Since Colonisation.” Animal Conservation 8: 407–413.

[ece372270-bib-0050] Phillips, B. L. , and R. Shine . 2006. “An Invasive Species Induces Rapid Adaptive Change in a Native Predator: Cane Toads and Black Snakes in Australia.” Proceedings of the Royal Society B: Biological Sciences 273: 1545–1550. 10.1098/rspb.2006.3479 PMC1560325 16777750

[ece372270-bib-0051] Reichard, M. , K. Douda , M. Przybylski , et al. 2015. “Population‐Specific Responses to an Invasive Species.” Proceedings of the Royal Society B: Biological Sciences 282: 1063. 10.1098/rspb.2015.1063 PMC4528524 26180070

[ece372270-bib-0052] Reichard, M. , H. Liu , and C. Smith . 2007. “The Co‐Evolutionary Relationship Between Bitterling Fishes and Freshwater Mussels: Insights From Interspecific Comparisons.” Evolutionary Ecology Research 9: 239–259.

[ece372270-bib-0053] Reichard, M. , M. Ondracková , M. Przybylski , H. Liu , and C. Smith . 2006. “The Costs and Benefits in an Unusual Symbiosis: Experimental Evidence That Bitterling Fish ( *Rhodeus sericeus* ) Are Parasites of Unionid Mussels in Europe.” Journal of Evolutionary Biology 19: 788–796. 16674575 10.1111/j.1420-9101.2005.01051.x

[ece372270-bib-0054] Reichard, M. , M. Polačik , A. S. Tarkan , et al. 2010. “The Bitterling‐Mussel Coevolutionary Relationship in Areas of Recent and Ancient Sympatry.” Evolution 64: 3047–3056. 20482611 10.1111/j.1558-5646.2010.01032.x

[ece372270-bib-0055] Reichard, M. , M. Przybylski , P. Kaniewska , H. Liu , and C. Smith . 2007. “A Possible Evolutionary Lag in the Relationship Between Freshwater Mussels and European Bitterling.” Journal of Fish Biology 70: 709–725.

[ece372270-bib-0056] Reichard, M. , M. Vrtílek , K. Douda , and C. Smith . 2012. “An Invasive Species Reverses the Roles in a Host–Parasite Relationship Between Bitterling Fish and Unionid Mussels.” Biology Letters 8: 601–604. 22337503 10.1098/rsbl.2011.1234 PMC3391448

[ece372270-bib-0057] Rouchet, R. , C. Smith , H. Liu , et al. 2017. “Avoidance of Host Resistance in the Oviposition‐Site Preferences of Rose Bitterling.” Evolutionary Ecology 31: 769–783.

[ece372270-bib-0058] Roznik, E. A. , K. L. Surbaugh , N. Cano , and J. R. Rohr . 2020. “Elucidating Mechanisms of Invasion Success: Effects of Parasite Removal on Growth and Survival Rates of Invasive and Native Frogs.” Journal of Applied Ecology 57: 1078–1088. 33071307 10.1111/1365-2664.13634 PMC7566891

[ece372270-bib-0059] Sage, R. F. 2020. “Global Change Biology: A Primer.” Global Change Biology 26: 3–30. 31663217 10.1111/gcb.14893

[ece372270-bib-0060] Saul, W.‐C. , and J. M. Jeschke . 2015. “Eco‐Evolutionary Experience in Novel Species Interactions.” Ecology Letters 18: 236–245. 25626585 10.1111/ele.12408

[ece372270-bib-0061] Shine, R. 2012. “Invasive Species as Drivers of Evolutionary Change: Cane Toads in Tropical Australia.” Evolutionary Applications 5: 107–116. 25568034 10.1111/j.1752-4571.2011.00201.x PMC3353345

[ece372270-bib-0062] Simberloff, D. , J.‐L. Martin , P. Genovesi , et al. 2013. “Impacts of Biological Invasions: What's What and the Way Forward.” Trends in Ecology & Evolution 28: 58–66. 22889499 10.1016/j.tree.2012.07.013

[ece372270-bib-0063] Smith, C. , A. Douglas , and P. Jurajda . 2002. “Sexual Conflict, Sexual Selection and Sperm Competition in the Spawning Decisions of Bitterling, *Rhodeus sericeus* .” Behavioral Ecology and Sociobiology 51: 433–439.

[ece372270-bib-0064] Smith, C. , M. Reichard , P. Jurajda , and M. Przybylski . 2004. “The Reproductive Ecology of the European Bitterling ( *Rhodeus sericeus* ).” Journal of Zoology 262: 107–124.

[ece372270-bib-0065] Smith, C. , K. Rippon , A. Douglas , and P. Jurajda . 2001. “A Proximate Cue for Oviposition Site Choice in the Bitterling ( *Rhodeus sericeus* ).” Freshwater Biology 46: 903–911.

[ece372270-bib-0066] Smith, C. , M. Warren , R. Rouchet , and M. Reichard . 2014. “The Function of Multiple Ejaculations in Bitterling.” Journal of Evolutionary Biology 27: 1819–1829. 24925267 10.1111/jeb.12432

[ece372270-bib-0067] Spence, R. , M. Reichard , and C. Smith . 2013. “Strategic Sperm Allocation and a Coolidge Effect in an Externally Fertilizing Species.” Behavioral Ecology 24: 82–88.

[ece372270-bib-0068] Stewart, P. S. , R. A. Hill , P. A. Stephens , M. J. Whittingham , and W. Dawson . 2021. “Impacts of Invasive Plants on Animal Behaviour.” Ecology Letters 24: 891–907. 33524221 10.1111/ele.13687

[ece372270-bib-0069] Strauss, S. Y. , J. A. Lau , and S. P. Carroll . 2006. “Evolutionary Responses of Natives to Introduced Species: What Do Introductions Tell Us About Natural Communities?” Ecology Letters 9: 357–374. 16958902 10.1111/j.1461-0248.2005.00874.x

[ece372270-bib-0070] Strayer, D. L. , V. T. Eviner , J. M. Jeschke , and M. L. Pace . 2006. “Understanding the Long‐Term Effects of Species Invasions.” Trends in Ecology & Evolution 21: 645–651. 16859805 10.1016/j.tree.2006.07.007

[ece372270-bib-0071] Stuart, Y. E. , T. S. Campbell , P. A. Hohenlohe , R. G. Reynolds , L. J. Revell , and J. B. Losos . 2014. “Rapid Evolution of a Native Species Following Invasion by a Congener.” Science 346: 463–466. 25342801 10.1126/science.1257008

[ece372270-bib-0072] Taraschewski, H. 2006. “Hosts and Parasites as Aliens.” Journal of Helminthology 80: 99–128. 16768855 10.1079/joh2006364

[ece372270-bib-0073] Thompson, J. N. 1994. The Coevolutionary Process. University of Chicago press.

[ece372270-bib-0074] Thompson, J. N. 2010. “Four Central Points About Coevolution.” Evolution: Education and Outreach 3: 7–13.

[ece372270-bib-0075] Torchin, M. E. , K. D. Lafferty , A. P. Dobson , V. J. McKenzie , and A. M. Kuris . 2003. “Introduced Species and Their Missing Parasites.” Nature 421: 628–630. 12571595 10.1038/nature01346

[ece372270-bib-0076] Torchin, M. E. , K. D. Lafferty , and A. M. Kuris . 2001. “Release From Parasites as Natural Enemies: Increased Performance of a Globally Introduced Marine Crab.” Biological Invasions 3: 333–345.

[ece372270-bib-0077] Traveset, A. , and D. M. Richardson . 2006. “Biological Invasions as Disruptors of Plant Reproductive Mutualisms.” Trends in Ecology & Evolution 21: 208–216. 16701087 10.1016/j.tree.2006.01.006

[ece372270-bib-0078] U.S. Fish & Wildlife Service . 2019. Ecological Risk Screening Summary—Bitterling (Rhodeus sericeus). U.S. Fish & Wildlife Service.

[ece372270-bib-0079] Vilà, M. , C. Basnou , P. Pyšek , et al. 2009. “How Well Do We Understand the Impacts of Alien Species on Ecosystem Services? A Pan‐European, Cross‐Taxa Assessment.” Frontiers in Ecology and the Environment 8: 135–144.

[ece372270-bib-0080] Whitney, K. D. , and C. A. Gabler . 2008. “Rapid Evolution in Introduced Species, ‘Invasive Traits’ and Recipient Communities: Challenges for Predicting Invasive Potential.” Diversity and Distributions 14: 569–580.

[ece372270-bib-0081] Yi, W. , M. Reichard , M. Rücklin , and M. K. Richardson . 2024. “Parasitic Fish Embryos Do a “Front‐Flip” on the Yolk to Resist Expulsion From the Host.” Proceedings of the National Academy of Sciences 121: e2310082121. 10.1073/pnas.2310082121 PMC10907307 38377205

[ece372270-bib-0082] Zangerl, A. R. , and M. R. Berenbaum . 2003. “Phenotype Matching in Wild Parsnip and Parsnip Webworms: Causes and Consequences.” Evolution 57: 806–815. 12778550 10.1111/j.0014-3820.2003.tb00292.x

[ece372270-bib-0083] Zdanowski, B. 1996. Nieznana szczeżuja (Anodonta sp.) w podgrzanych Jeziorach Konińskich. XII‐th Krajowe Seminarium Malakologiczne.

