# Supplementary material for: Temporal Dynamics of Biological Invasions: Perception of Host Quality Differs Between Native and Alien Host Species
Source: Ecol Evol. 2025 Oct 6;15(10):e72270. doi: 10.1002/ece3.72270 (PMC12497942; doi:10.1002/ece3.72270)
Supplement: Supplementary file 1 — Appendix S1: Table S1. Appendix S1: Table S2. [file ECE3-15-e72270-s001.docx]

**Appendix S1:**

**Table S1.** Results of Generalised Linear Mixed Models with Tweedie distribution on (a) male ejaculation frequency and (b) female skimming frequency to test the Population Preference Hypothesis in *Anodonta anatina*. Estimated coefficients (and their 95% confidence interval and statistical significance of their contrast with reference treatment: mussel with recent association, fish without association) for a combination of *A. anatina* mussel origin treatment (recent, intermediate, old association with between *S. woodiana* and bitterling fish) and bitterling origin treatment (fish from populations without, with recent, intermediate and old association with *S. woodiana* mussels).

|  | 1. Male ejaculation | | | 1. Female skimming | | |
| --- | --- | --- | --- | --- | --- | --- |
| *Coefficient* | *Estimates* | *Conf. Int.* | *P* | *Estimates* | *Conf. Int* | *P-value* |
| Intercept(recent) | 2.01 | 1.57 – 2.45 | <0.001 | 0.20 | -0.47 – 0.87 | 0.561 |
| Mussel (intermed) | -0.15 | -0.61 – 0.32 | 0.542 | 0.12 | -0.46 – 0.70 | 0.683 |
| Mussel (old) | -0.07 | -0.53 – 0.39 | 0.767 | -0.22 | -0.83 – 0.39 | 0.475 |
| Fish (recent) | -0.42 | -0.95 – 0.12 | 0.127 | -0.38 | -1.21 – 0.45 | 0.364 |
| Fish (intermed) | -0.39 | -0.92 – 0.14 | 0.146 | -0.33 | -1.18 – 0.52 | 0.443 |
| Fish (old) | -0.27 | -0.79 – 0.25 | 0.311 | -0.24 | -1.06 – 0.58 | 0.566 |
| Random Effects | | | | | | |
| σ^2^ (τ_00_ _replicateID_) | 1.18 (0.00) | | | 1.58 (0.54) | | |
| Marg R2 / Cond R2 | 0.026 / NA | | | 0.019 / 0.270 | | |
| N_obs_ (N_replicateID_) | 240 (80) | | | 240 (80) | | |

**Appendix S2:**

**Table S2.** Results of Generalised Linear Mixed Models with Tweedie distribution on (a) male ejaculation frequency and (b) female skimming frequency to test the Strong Coevolution Hypothesis in *Anodonta anatina*. Estimated coefficients (and their 95% confidence interval and statistical significance of their contrast with reference treatment: allopatric relationship with *A. anatina* and recent bitterling – *S. woodiana* association) for a combination of *A. anatina* origin (allopatric or sympatric with *S. woodiana* population) and bitterling origin treatment (fish from populations with recent, intermediate and old association with *S. woodiana* mussels).

|  | 1. Male ejaculation | | | 1. Female skimming | | |
| --- | --- | --- | --- | --- | --- | --- |
| *Coefficient* | *Estimates* | *Conf. Int.* | *P* | *Estimates* | *Conf. Int* | *P-value* |
| Intercept (allopatric) | 1.48 | 0.98 – 1.99 | <0.001 | -0.10 | -0.80 – 0.60 | 0.786 |
| Sympatry (sympatric) | -0.34 | -0.80 – 0.13 | 0.162 | -0.62 | -1.26 – 0.03 | 0.060 |
| Fish (intermed) | -0.01 | -0.64 – 0.63 | 0.980 | -0.03 | -0.96 – 0.89 | 0.943 |
| Fish (old) | 0.18 | -0.44 – 0.80 | 0.570 | 0.12 | -0.78 – 1.01 | 0.798 |
| Random Effects | | | | | | |
| σ^2^ (τ_00_ _replicateID_) | 1.15 (0.29) | | | 1.60 (0.79) | | |
| Marg R2 / Cond R2 | 0.022 / 0.218 | | | 0.036 / 0.355 | | |
| N_obs_ (N_replicateID_) | 180 (60) | | | 180 (60) | | |
